# Memorandum of Two Cases of Cæsarean Section, Performed at the Civil Hospital, Prague

**Published:** 1827-11

**Authors:** 


### CÆSAREAN SECTION.

*Memorandum of two Cases of Cæsarean Section, performed at the* Civil Hospital, Prague. Communicated in a Letter from Dr. Lefevre to Mr. Travers.

During my stay at Prague, I had an opportunity of seeing two women who had undergone and recovered from the Cæsarean operation. The one was a Jewess, very much deformed by curvature of the spine, and of very small stature. She was pregnant three years ago for the first time, and was twenty-seven years of age. She was three days in labour, and had no other assistance than that of a midwife, who did not even attempt to deliver her by means of instruments. On the fourth day she was conveyed to the hospital, and, as the pelvis was found on examination to be excessively contracted, the Cæsarean operation was immediately determined upon, and performed by Dr. Fridzt, surgeon and clinical professor to the Civil Hospital at Prague. The operation was not followed by any serious consequences, and the patient was dismissed cured. Upon examining the woman by permission of the professor, I found a cicatrix extending from the umbilicus to the symphysis pubis. As the body was much inclined towards the left side, so the cicatrix was of a semilunar form, and the integuments of the abdomen were plaited as it were in large folds. It is more than three years since the operation was performed; the woman is in perfect health, and gains her bread by labour. She complains only occasionally of pain and numbness in both thighs.

The second case was still in the hospital, and had been operated upon eight weeks previous to my seeing her. She is the wife of a Bohemian peasant, and has had one child,—the labour was perfectly natural. She became again pregnant, and gestation went on naturally till the end of the sixth month, except that the tumor of the abdomen was more projecting than usual, and inclined towards the right side. After the sixth month, the movements of the child were not sensible; the breasts became enlarged, and milk oozed from them for a short time. There was also slight uterine hemorrhage. At the end of the ninth month, labour pains came on, were very severe, but subsided, and returned at intervals of ten days till the end of the eleventh month. She was at this time taken to the hospital, and examined by Dr. Krombohoff, professor of midwifery in the university of Prague. He found a spheroidal tumor in the vagina, and the os uteri turned towards the left side. The case was pronounced one of extra-uterine pregnancy, and the woman was conveyed into the surgical wards, and operated upon by Dr. Fridzt. The incision was six inches in length, extending from half an inch below the umbilicus to the symphysis pubis. The head of the child, which presented, was extracted by a simple manœuvre. The placenta and membranes were left in the abdomen. The child had been dead apparently some time. The wound was united by means of adhesive plaster, about an inch of the inferior portion being left open for the escape of fluids from the abdomen. These were the only means employed. She was once bled from the arm, and leeches were once applied. The wound is now almost completely healed, a small fistulous opening remaining only at the inferior portion, from which a healthy pus is secreted. She is now taking tonics, and is allowed meat, but neither wine nor beer. The functions are all becoming natural. Stools have hitherto been procured only by means of enemas. The countenance is animated and cheerful; the pulse small, but not irritable; the appetite good. I saw the woman three times during my stay at Prague, and there is every probability of her speedy recovery. The name written on the board at the head of the bed was "Pototza Veronica, æt. thirty-two, operat. 10 July." I requested Dr. Fridzt to allow me to have a statement of the case in writing, to which he most kindly consented, and to which he has subscribed his name.

I inclose it as it was drawn up by a German student: it contains merely what I have written. This is the third operation performed at this hospital by the same professor: one of these only had failed.

*Prague; September 1st, 1827.*

---

